# Regulation of RIG-I Activation by K63-Linked Polyubiquitination

**DOI:** 10.3389/fimmu.2017.01942

**Published:** 2018-01-05

**Authors:** Masaaki Okamoto, Takahisa Kouwaki, Yoshimi Fukushima, Hiroyuki Oshiumi

**Affiliations:** ^1^Faculty of Life Sciences, Department of Immunology, Graduate School of Medical Sciences, Kumamoto University, Kumamoto, Japan; ^2^PRESTO, Japan Science and Technology Agency, Kumamoto, Japan

**Keywords:** RIG-I, ubiquitin, innate immunity, virus, signaling pathway

## Abstract

RIG-I is a pattern recognition receptor and recognizes cytoplasmic viral double-stranded RNA (dsRNA). Influenza A virus, hepatitis C virus, and several other pathogenic viruses are mainly recognized by RIG-I, resulting in the activation of the innate immune responses. The protein comprises N-terminal two caspase activation and recruitment domains (2CARDs), an RNA helicase domain, and the C-terminal domain (CTD). The CTD recognizes 5′-triphosphate viral dsRNA. After recognition of viral dsRNA, the protein harbors K63-linked polyubiquitination essential for RIG-I activation. First, it was reported that TRIM25 ubiquitin ligase delivered K63-linked polyubiquitin moiety to the 2CARDs. The polyubiquitin chain stabilizes a structure called the 2CARD tetramer, in which four 2CARDs assemble and make a core that promotes the aggregation of the mitochondrial antiviral-signaling (MAVS) protein on mitochondria. MAVS aggregation then triggers the signal to induce the innate immune responses. However, subsequent studies have reported that Riplet, MEX3C, and TRIM4 ubiquitin ligases are also involved in K63-linked polyubiquitination and the activation of RIG-I. MEX3C and TRIM4 mediate polyubiquitination of the 2CARDs. By contrast, Riplet ubiquitinates the CTD. The physiological significance of each ubiquitin ligases has been shown by knockout and knockdown studies, but there appears to be contradictory to evidence reported in the literature. In this review, we summarize recent findings related to K63-linked polyubiquitination and propose a model that could reconcile current contradictory theories. We also discuss the physiological significance of the ubiquitin ligases in the immune system against viral infection.

## Introduction

Pattern recognition receptors (PRRs) recognize viral nucleic acids and trigger a signal to induce the innate immune responses during viral infection (1, 2). RIG-I is a cytoplasmic RNA helicase and a PRR that recognizes cytoplasmic 5′ tri- or diphosphate double-stranded RNA (dsRNA) (3–5). RIG-I binds relatively short dsRNA (<1 kbp) and is involved in the recognition of various viral infections, such as influenza A and B viruses, Japanese encephalitis virus, hepatitis C virus (HCV), dengue virus, and West Nile virus (6–8). After recognition of viral RNA, RIG-I associates with an adaptor protein, mitochondrial antiviral-signaling (MAVS) protein, also called IPS-1, Cardif, and VISA (9–12), resulting in the aggregation of MAVS on the outer membrane of mitochondria. This aggregation triggers a signal to induce the expression of type I interferon (IFN) and other inflammatory cytokines (13).

The RIG-I protein comprises two caspase-activation and recruitment domains (2CARDs) at the N-terminal region, an RNA helicase domain, and a C-terminal domain (CTD) (14–16). Viral dsRNA binds to the RNA helicase domain and the CTD, and 5′ tri- and diphosphate are recognized by the CTD (16, 17). The N-terminal 2CARDs are responsible for the association with MAVS and, therefore, are required for triggering downstream signaling (5). In resting cells, the C-terminal region, which includes the CTD and the linker region between the CTD and the helicase domain, suppresses the N-terminal 2CARDs (14, 18). Binding of the CTD to dsRNA induces the conformational change of the RIG-I protein, resulting in the release of the 2CARDs (14). Subsequently, the proteins assemble along viral dsRNA and form a nucleoprotein filament (19). The released 2CARDs also assemble and form a 2CARD tetramer structure (20). The structure functions as a core for MAVS aggregation on mitochondria (21).

The RIG-I protein harbors Lys 63-linked (K63-linked) polyubiquitination required for its activation (22). TRIM25 is a ubiquitin ligase and delivers K63-linked polyubiquitin moiety to the RIG-I 2CARDs (22, 23). The polyubiquitin chains stabilize the 2CARD tetramer structure (21). The physiological significance of TRIM25 in RIG-I activation has been shown by several studies (22–27). However, recent studies have reported three other ubiquitin ligases, RING finger protein leading to RIG-I activation (Riplet), mex-3 RNA-binding family member C (MEX3C), and TRIM4, which are required for the polyubiquitination and activation of RIG-I (28–30).

## Polyubiquitination of the 2CARDs of RIG-I

Ubiquitin ligases add a ubiquitin chain at K but not R residues of the target protein. There are 18 K residues in the RIG-I 2CARDs, and mass spectrometry analysis revealed that the 2CARDs fragment carried K63-linked polyubiquitin chains at K99, K169, K172, K181, K190, and K193 (22). Knockdown of TRIM25 abrogated polyubiquitination of the 2CARDs fragment, suggesting that TRIM25 mediates K63-linked polyubiquitination at the 6 K residues of the 2CARDs (22). The 2CARDs fragment has an ability to bind to MAVS, and overexpression of the 2CARDs fragment leads to auto-activation of signaling (5, 9–12). An amino acid substitution assay revealed that the substitution of K172, but not of other K residues, with R abrogated the signaling induced by the 2CARDs fragment (22). Knockout of TRIM25 severely reduced RIG-I-mediated type I IFN production during viral infection. These observations indicate the importance of TRIM25-mediated K172 ubiquitination (22).

Evidence also suggests that TRIM25 produces unanchored K63-linked polyubiquitin chains in response to viral infection and delivered them to RIG-I (23). The same study also showed that the K172 residue of RIG-I was important for non-covalent binding of RIG-I with unanchored polyubiquitin chains (23). Considering that mass spectrometry analysis revealed the covalent binding of RIG-I with K63-linked polyubiquitin chains, these observations indicate that either covalent or non-covalent binding with polyubiquitin chains is sufficient for RIG-I 2CARDs activation (23, 26). A structural study of RIG-I 2CARDs tetramer provided evidence that both covalent and non-covalent binding of polyubiquitin chains promotes the formation of the 2CARD tetramer structure (21, 26). The TRIM25 activity itself is regulated by the physical interaction between the TRIM25 SPRY domain and RIG-I 2CARDs (31). The cooperative assembly of TRIM25 and RIG-I facilitates the dimerization of the TRIM25 RING domain, which is required for TRIM25 to make polyubiquitin chain (31).

**Figure 1:**
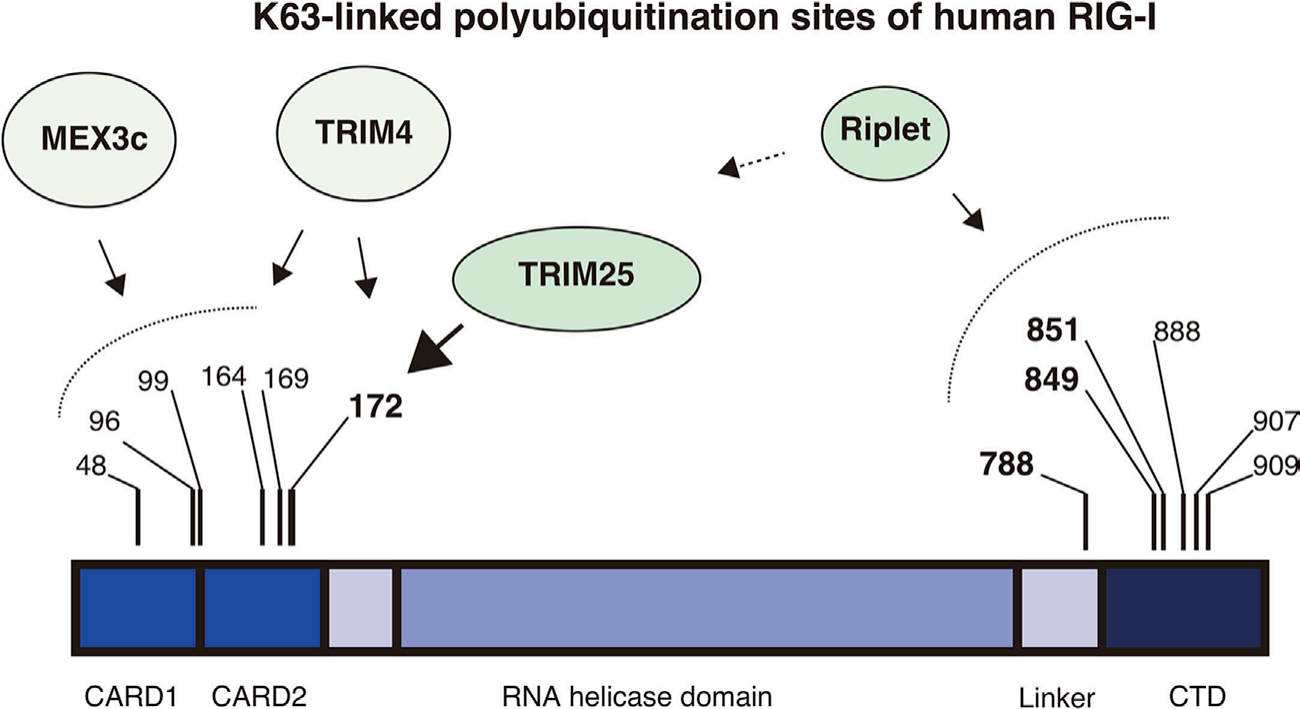
K63-linked polyubiquitination sites of RIG-I. The RIG-I protein harbors K63-linked polyubiquitination at multiple sites. The protein comprises the N-terminal 2CARDs, the RNA helicase domain, and the C-terminal domain (CTD). The four ubiquitin ligases, Riplet, TRIM25, MEX3C, and TRIM4 are involved in the ubiquitination. Mass spectrometry analyses showed that Lys 172 of the 2CARDs carries K63-linked polyubiquitination. TRIM25 mediates K63-linked polyubiquitination at Lys 172 of the 2CARDs or deliver unanchored K63-linked polyubiquitin chains to it. MEX3C mediates K63-linked polyubiquitination at Lys 48, 99, and 169. TRIM4 mediates it at Lys 164 and 172. Shi et al. reported that Lys 96 of the 2CARDs carried K63-linked polyubiquitin chain and were required for RIG-I activation. By contrast, the Riplet ubiquitin ligase mediates K63-linked polyubiquitination of the CTD and the linker region between the CTD and the RNA helicase domain. Lys 788, 849, and 851 are important for RIG-I activation, but other Lys residues, including Lys 888, 907, and 909, also harbor ubiquitination.

An accumulating body of evidence has shown that TRIM25 delivers K63-linked polyubiquitin moiety to RIG-I 2CARDs for RIG-I activation and that the K172 residue is important for the binding of RIG-I to polyubiquitin chains (26, 27, 32). However, subsequent studies revealed that not only K172 but also other K residues are also important for the binding of RIG-I to K63-linked polyubiquitin chains. First, Shigemoto et al. reported that the expression of the RIG-I K172R full-length protein could compensate for a defect in RIG-I knockout mouse embryonic fibroblasts (MEFs) after Sendai virus infection (33). Second, two other ubiquitin ligases, MEX3C and TRIM4, were reported to mediate polyubiquitination of the RIG-I 2CARDs at other K residues (29, 30). Kuniyoshi et al. reported that MEX3C-mediated polyubiquitination was reduced by mutations at K48, K99, and K169, and Yan et al. reported that TRIM4 targeted K164 and K172 (30). Mass spectrometry analysis has also revealed the ubiquitination at K48, K96, K170, as well as K172 and K190 of the 2CARDs (34). They reported that simultaneous amino acids substitutions at K48, K96, and K172 substantially reduced the polyubiquitination of RIG-I (34). These observations suggest that K63-linked polyubiquitination at these K residues can compensate for the loss of K172 binding to the ubiquitin chain under certain conditions (Figure 1).

## Polyubiquitination of the RIG-I C-Terminal Region

Riplet, another ubiquitin ligase, is also involved in K63-linked polyubiquitination and activation of RIG-I. Riplet was first isolated by our yeast two-hybrid screening using the RIG-I CTD fragment as a bait (28). An immunoprecipitation assay then confirmed that the protein bound to the CTD fragment, and our studies also indicated that Riplet mediates K63-linked polyubiquitination of the RIG-I CTD (28). Our mutation analysis indicated that K849 and K851 of the CTD were important for Riplet-mediated polyubiquitination, and Riplet also targeted other K residues, including K888, K907, and K909 of the CTD and K788 in the linker region between the CTD and the helicase domain (24, 28) (Figure 1).

To assess the physiological significance of the protein, we generated Riplet knockout mice. Knockout of Riplet severely impaired the type I IFN and IL-6 production in MEF, macrophages, and conventional dendritic cells following influenza A virus and vesicular stomatitis virus (VSV) infections. In addition, Riplet KO mice were susceptible to VSV infection compared with wild-type mice (35). These observations indicate that Riplet-mediated polyubiquitination of RIG-I is essential for RIG-I activation during viral infection *in vivo*.

**Figure 2:**
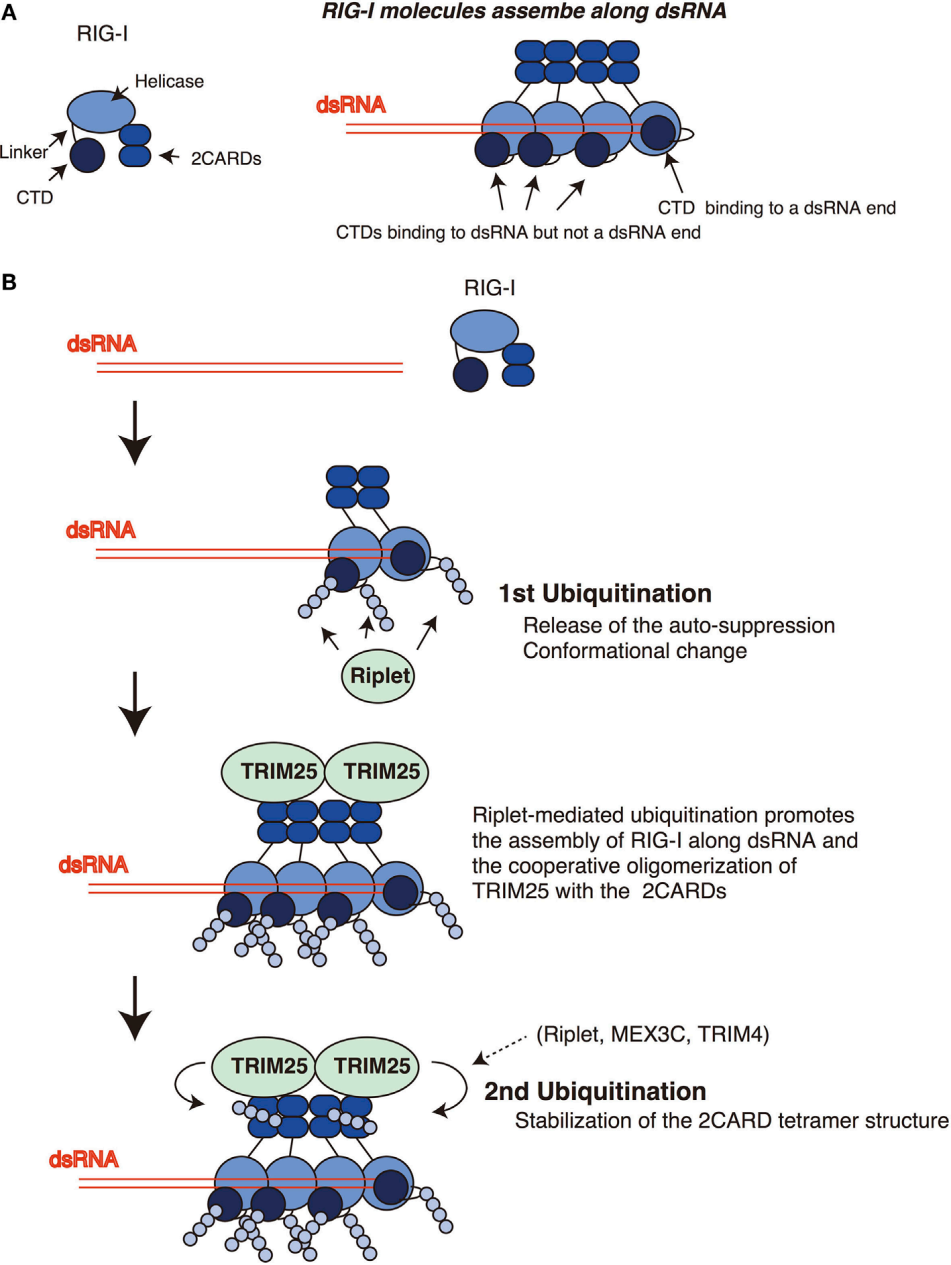
Sequential ubiquitination model for RIG-I. **(A)** The RIG-I proteins assemble along double-stranded RNA (dsRNA). The C-terminal domain (CTD) recognizes 5′ triphosphate at the end of dsRNA. One CTD is required for the recognition of 5′ triphosphate, whereas other CTDs of RIG-I molecules that assemble along dsRNA do not bind the end of dsRNA. **(B)** Riplet mediates K63-linked polyubiquitination of the linker region of RIG-I associated with dsRNA. In addition, Riplet can ubiquitinate the CTDs that assemble along dsRNA but not at the end of dsRNA. The linker region is responsible for the auto-suppression of the 2CARDs, and the CTD activation promotes the conformational change of the RIG-I protein. Riplet-mediated polyubiquitination of the C-terminal region (first ubiquitination) is expected to promote the release of the auto-suppression and/or the assembly of RIG-I along dsRNA *via* conformational change, leading to the binding of TRIM25 to the 2CARDs. Subsequently, cooperative oligomerization of TRIM25 with the 2CARDs induces the TRIM25-mediated polymerization of ubiquitin (second ubiquitination) required for stabilizing the 2CARD tetramer structure.

An RIG-I C-terminal fragment (735–925 aa region), which includes the linker region and the CTD, suppresses the 2CARD activation (14), and Kageyama et al. reported that the linker region (746–801 aa) was responsible for the auto-suppression (18). Riplet targets the K788 in the linker region. Therefore, it is possible that the ubiquitination of the linker region disrupts the auto-suppression. The structure analysis revealed that K849, K851, K858, and K888 of the CTD bind to 5′ triphosphate of dsRNA ends (36). The K849, K851, and K888 of the CTD are targeted by Riplet. Binding of the CTD to 5′ triphosphate of dsRNA was reported to induce conformational change of the RIG-I protein (16). Although several RIG-I molecules assemble along dsRNA, only one RIG-I molecule binds the 5′ triphosphate at the dsRNA end (19) (Figure 2A). Therefore, Riplet could access the K849, K851, and K888 of the CTDs of the RIG-I molecules associating with dsRNA (but not the end of dsRNA) to induce conformational change of RIG-I. These K residues are located at the edge of the CTD basic cleft, which is an RNA-binding site (16). Further studies are required to reveal underlying mechanism of Riplet-mediated RIG-I activation.

## Sequential Ubiquitination Model

Despite the identification of four ubiquitin ligases, we found that knockout of Riplet alone could abolish the polyubiquitination of the endogenous RIG-I protein (24, 35). Recently, Shi et al. also reported that knockout of Riplet is sufficient to abolish the polyubiquitination of RIG-I and, therefore, claimed that Riplet is a primary ubiquitin ligase and mediates K63-linked polyubiquitination of the 2CARDs (34). However, their model appears to be contradict to previous papers showing that TRIM25 plays a crucial role in RIG-I activation.

Previously, we have postulated a sequential ubiquitination model that Riplet-mediated polyubiquitination of RIG-I C-terminal region is a prerequisite for the polyubiquitination of the 2CARDs (Figure 2) (24). This model could explain the apparent discrepancy in the literature, because due to the initial failure to polyubiquitinate the C-terminal region, this would obstruct the subsequent polyubiquitination of the 2CARDs by other ubiquitin ligases. This indicates that knockout of Riplet alone is sufficient to abolish the polyubiquitination of the CTD, the linker region, and the 2CARDs. In a previous study, we have shown that Riplet promotes the binding of TRIM25 to RIG-I (24). This observation supports the sequential model. It is expected that Riplet-mediated polyubiquitination leads to the release of auto-suppression and/or conformational change of RIG-I, which would allow the access of TRIM25 to the 2CARDs and/or promote RIG-I assembly along dsRNA (Figure 2B). Considering that higher-order oligomerization of TRIM25 with the 2CARDs is required to induce TRIM25-mediated polyubiquitination (31), it is not surprising that Riplet-mediated C-terminal ubiquitination is a prerequisite for the second ubiquitination by TRIM25 (Figure 2B).

MEX3C or TRIM4 might compensate for the defect of TRIM25 under certain experimental conditions, because these two ubiquitin ligases target the 2CARDs in a similar way (Figure 1). Although we failed to detect an interaction between Riplet and the 2CARDs fragment, other groups have reported that Riplet bound to the 2CARDs fragment and was involved in the K63-linked polyubiquitination of the 2CARDs (34, 37). These observations do not conflict with the sequential ubiquitination model because several ubiquitin ligases can compensate for the loss of TRIM25 in some conditions. We do not exclude the possibility that Riplet is not only involved in the primary ubiquitination of the CTD and the linker region but also the secondary ubiquitination of the 2CARDs (Figure 2).

## Viral Targeting of Ubiquitin Ligases

Type I IFN exhibits a strong antiviral effect, and hence several viruses have evolved to suppress the type I IFN production. HCV is a major cause of hepatocellular carcinoma and persistently infects hepatocytes over several decades without exclusion by the host immune system. A viral NS3-4A protease is required to cleave viral polypeptides and produce mature viral proteins; however, it is also important to suppress the host innate immune response. NS3-4A of HCV cleaves MAVS, which results in the release of MAVS from mitochondria (12). Several reports have shown that released MAVS protein fails to trigger signaling to induce type I IFN production (9). Accordingly, NS3-4A-mediated cleavage of MAVS abrogates RIG-I-mediated type I IFN production. NS3-4A protein also targets the Riplet protein. The RING finger domain is a catalytic domain of the Riplet protein, and viral NS3-4A protease cleaves the domain and destabilizes the protein (24). NS1 protein of influenza A virus also has the ability to suppress type I IFN production (38). Although several mechanisms have been postulated, Gack et al. reported that viral NS1 bound to TRIM25 and inhibited TRIM25-mediated polyubiquitination of RIG-I (39). In further study, they reported that NS1 protein also targeted the Riplet protein and inhibited RIG-I polyubiquitination (40). Severe acute respiratory syndrome coronavirus (SARS-CoV) also interferes TRIM25 function (41). The nucleocapsid protein of SARS-CoV physically interacted with TRIM25 and inhibited the binding of TRIM25 to RIG-I, resulting in the attenuation of RIG-I-signaling (41). These data imply that viruses obtained the ability to suppress the ubiquitin ligases to escape innate immune responses. Conversely, these data indicate the importance of the two ubiquitin ligases, TRIM25 and Riplet, for the antiviral innate immune response.

## Perspectives

TRIM25 is also called EFP, and it has been shown that TRIM25/EFP mediates the polyubiquitination of 14-3-3σ and promotes its proteolysis to suppress the growth of breast tumor cells (42). Although other targets of Riplet have not been reported, it was shown that mutations on human Riplet genes (also called RNF135) are linked to learning disabilities and several neuropsychiatric disorders (43, 44). Thus, it is expected that Riplet targets the proteins involved in these conditions. There are several reports that Riplet and TRIM25 are involved in tumorigenesis (42, 45, 46). As several viruses have the ability to abrogate the described ubiquitin ligases, it is expected that viral protein-mediated inhibition of the ubiquitin ligases affects both innate immunity and other phenomena, such as virus-induced tumorigenesis and neuropsychiatric disorders.

There are two protein families related to RIG-I called RIG-I-like receptors (RLRs). LGP2 is an RLR, and the CTD structure of the protein is similar to that of RIG-I (14). Initial studies reported that LGP2 is a negative regulator for RIG-I signaling (15, 47). However, knockout and biochemical studies have revealed that LGP2 functions as a positive regulator of the RIG-I pathway (48, 49). LGP2 is also expressed in CD8^+^ T cells and is required for CD8^+^ T cell proliferation (50). It remains unclear whether LGP2 carries a ubiquitin chain. Considering the conservation of the CTD between RIG-I and LGP2, it is possible that Riplet also targets the CTD of LGP2 and affects LGP2-mediated RIG-I activation and CD8^+^ T cell proliferation. Further studies are required to fully elucidate the role of the ubiquitin ligases in the antiviral immune response.

## Author Contributions

HO and MO wrote the manuscript. TK and YF helped the discussion.

## Conflict of Interest Statement

The authors declare that the research was conducted in the absence of any commercial or financial relationships that could be construed as a potential conflict of interest.
